# Effect of Denosumab on Bone Mineral Density and Markers of Bone Turnover among Postmenopausal Women with Osteoporosis

**DOI:** 10.1155/2016/8738959

**Published:** 2016-08-08

**Authors:** A. Sánchez, L. R. Brun, H. Salerni, P. R. Costanzo, D. González, A. Bagur, B. Oliveri, M. B. Zanchetta, V. Farías, L. Maffei, V. Premrou, J. L. Mansur, M. S. Larroudé, M. A. Sarli, P. Rey, M. R. Ulla, M. M. Pavlove, S. Karlsbrum, M. L. Brance

**Affiliations:** ^1^Centro de Endocrinología, Rosario, Argentina; ^2^Laboratorio de Biología Ósea, Facultad de Ciencias Médicas, Universidad Nacional de Rosario, Rosario, Argentina; ^3^Consultorios de Investigación Clínica Endocrinológica y del Metabolismo Óseo (CICEMO), Buenos Aires, Argentina; ^4^Mautalen Salud e Investigación, Buenos Aires, Argentina; ^5^Laboratorio Osteoporosis y Enfermedades Metabólicas Óseas, INIGEM, UBA-CONICET, Hospital de Clínicas, Buenos Aires, Argentina; ^6^Instituto de Diagnóstico e Investigaciones Metabólicas (IDIM), Cátedra de Osteología y Metabolismo Mineral, Universidad del Salvador, Buenos Aires, Argentina; ^7^Consultorios Asociados de Endocrinología Dra. Laura Maffei, Buenos Aires, Argentina; ^8^Centro de Endocrinología y Osteoporosis, La Plata, Argentina; ^9^Hospital Milstein, Buenos Aires, Argentina; ^10^Centro de Endocrinología y Osteopatías Médicas, Córdoba, Argentina; ^11^Hospital Durand, Buenos Aires, Argentina; ^12^Centro de Reumatología, Rosario, Argentina

## Abstract

The aim of this study was to evaluate the effect of denosumab (Dmab) on bone mineral density (BMD) and bone turnover markers after 1 year of treatment. Additionally, the effect of Dmab in bisphosphonate-naïve patients (BP-naïve) compared to patients previously treated with bisphosphonates (BP-prior) was analyzed. This retrospective study included 425 postmenopausal women treated with Dmab for 1 year in clinical practice conditions in specialized centers from Argentina. Participants were also divided according to previous bisphosphonate treatment into BP-naïve and BP-prior. A control group of patients treated with BP not switched to Dmab matched by sex, age, and body mass index was used. Data are expressed as mean ± SEM. After 1 year of treatment with Dmab the bone formation markers total alkaline phosphatase and osteocalcin were significantly decreased (23.36% and 43.97%, resp.), as was the bone resorption marker s-CTX (69.61%). Significant increases in BMD were observed at the lumbar spine, femoral neck, and total hip without differences between BP-naïve and BP-prior. A better BMD response was found in BP-prior group compared with BP treated patients not switched to Dmab.* Conclusion*. Dmab treatment increased BMD and decreased bone turnover markers in the whole group, with similar response in BP-naïve and BP-prior patients. A better BMD response in BP-prior patients versus BP treated patients not switched to Dmab was observed.

## 1. Introduction

Osteoporosis is a chronic condition characterized by decreased bone mass and deterioration of bone microarchitecture which compromises bone strength predisposing to fragility fractures. Current available treatments for osteoporosis are selective estrogen-receptor modulators, antiresorptive medications which include bisphosphonates (BP) and denosumab (Dmab), bone-forming agents such as parathyroid hormone (PTH_1–84_ or its fragment PTH_1–34_), and strontium ranelate (SrR) which has a dual mechanism of action [1].

Dmab is a human monoclonal antibody to the receptor activator of nuclear factor-*κ*B ligand (RANKL) that blocks its binding to RANK, inhibiting the development and activity of osteoclasts, thus decreasing bone resorption [2–4].

In previous studies, Dmab treatment for up to 8 years increased BMD significantly at the lumbar spine, total hip, and one-third radius compared with placebo and reduced the risk of vertebral and nonvertebral fractures in postmenopausal women with osteoporosis [2, 5–8]. Also it has been shown to decrease hip fractures by 62% in patients ≥75 years after 3 years of treatment [9].

BP also reduce bone resorption, but through a mechanism of action different from Dmab. Patients treated with BP for osteoporosis may require a switch to other therapies. Those patients who suffer adverse events while on BP or have contraindications to receive them are of particular interest. Interestingly, Dmab is effective in patients who have previously received BP [10–12]. Dmab has been also shown to achieve greater increases in BMD compared with oral alendronate in anatomic regions with different percentage of trabecular and cortical bone [10, 11]. This is important because osteoporotic fractures are due to loss of bone in both compartments. Also, in high risk subjects, Dmab led to greater gains in BMD than oral BP at the total hip (2.2 versus 0.8%), femoral neck (1.8 versus 0.3%), and lumbar spine (3.7 versus 1.4%) [12].

The aim of this study was to evaluate the effect of Dmab on BMD and bone turnover markers after 1 year of treatment in clinical practice conditions in specialized centers from Argentina. Additionally, we ascertained the effect of Dmab in BP-naïve patients compared to patients previously treated with BP (BP-prior).

## 2. Patients and Methods

This retrospective study analyzed records from 425 postmenopausal women treated with Dmab (60 mg subcutaneously every 6 months) for 1 year in bone clinics from Argentina. All women had either a *T*-score of less than −2.5 at the hip or spine or a *T*-score of less than −2.0 plus other risk factors for fracture. All patients simultaneously received calcium (1,000 mg/day) and vitamin D (800 U/day). Women were excluded if they had medical conditions or took medications associated with bone disease. Patients were also analyzed considering the previous use of BP and were divided in two groups: BP-naïve (*n* = 61) and BP-prior (*n* = 269); 95 patients were not included in this analysis because they had received teriparatide or strontium ranelate previously (*n* = 68) and/or due to insufficient data about previous treatments (*n* = 27). A control group of patients treated with BP and followed up in the same clinics with similar inclusion and exclusion criteria but without receiving Dmab (*n* = 148) was analyzed. This control group was matched by sex, age, and body mass index (BMI) with the BP-prior group.

Weight (kg) and height (m) as anthropometric parameters were recorded to calculate body mass index (BMI) according to the following formula: BMI = weight/height^2^ (kg/m^2^).

BMD (g/cm^2^) was measured by dual-energy X-ray absorptiometry (DXA) with GE Lunar Prodigy equipment (GE Lunar, Madison, WI, USA) at the lumbar spine (L2–L4), femoral neck, and total hip. The coefficient of variation was less than 3% in all centers where the densitometries were performed.

Plasma calcium levels (mg/dL), plasma phosphate levels (mg/dL), and total alkaline phosphatase (tAP, UI/l) were spectrophotometrically measured. Serum parathyroid hormone (iPTH, pg/mL) was measured by chemiluminescent assay (iPTH Siemens Medical Solutions Diagnostics). Total serum 25-hydroxyvitamin D levels (25(OH)D, ng/mL) and serum carboxy-terminal crosslinking telopeptide of type I collagen (s-CTX, ng/L) were measured by electrochemiluminescence assay (Elecsys® Total Vitamin D Roche and Elecsys® *β*-CrossLaps Roche Diagnostics, resp.). Serum osteocalcin (BGP, ng/mL) was determined by electrochemiluminescence assay (Roche Diagnostics).

### 2.1. Data Analysis

Data are expressed as mean ± SEM and were analyzed with the Mann-Whitney test or Wilcoxon's signed rank test as appropriate. The Kolmogorov-Smirnov test for normality was used to assess the distribution of the data. Differences were considered significant if *p* < 0.05. Statistical analyses were performed with GraphPad Prism 2.0 (GraphPad, San Diego, USA).

## 3. Results

### 3.1. Subjects and Baseline Clinical Characteristics

Medical records from 425 postmenopausal women were analyzed. The main characteristics of the study population are shown in Table 1. No patient had to interrupt treatment with Dmab due to adverse effects.

### 3.2. Change in Bone Markers after Dmab Treatment

After 1 year of treatment with Dmab, the bone formation markers tAP (basal: 149.40 ± 5.65, versus 1 year: 114.50 ± 5.08 UI/L) and BGP (basal: 19.33 ± 0.75, versus 1 year: 10.83 ± 0.62 ng/mL) were significantly decreased (Wilcoxon's signed rank test, *p* < 0.0001), with a mean decline of 23.36% and 43.97%, respectively. Meanwhile, the bone resorption marker s-CTX (basal: 332.4 ± 14.39 versus 1 year: 101.00 ± 7.20 ng/L) significantly decreased by 69.61% (Wilcoxon's signed rank test, *p* < 0.0001).

### 3.3. Change in BMD after Dmab Treatment

**Figure 1:**
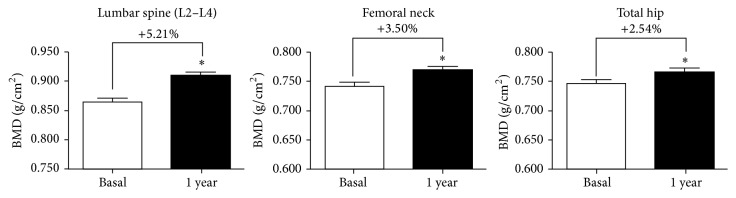
Change in BMD after 1 year of treatment with Dmab. *∗* indicates significant differences from basal (*p* < 0.0001).

After 1 year of treatment with Dmab an increase in BMD was observed in all regions (Wilcoxon's signed rank test, *p* < 0.0001): lumbar spine (LS): basal: 0.864 ± 0.006; 1 year: 0.909 ± 0.006; +5.21%, femoral neck (FN): basal: 0.742 ± 0.006, 1 year: 0.768 ± 0.007, +3.50%; and total hip (TH): basal: 0.747 ± 0.006; 1 year: 0.766 ± 0.006, +2.54% (Figure 1).

Those patients whose densitometric gain was ≥3% (the least significant change) after one year of Dmab treatment were considered “responders”: 65.2% of responders were found at the lumbar spine, 62.9% at the femoral neck, and 47.4% at the total hip.

### 3.4. Bisphosphonate-Naïve versus Bisphosphonate-Prior Patients

#### 3.4.1. Main Characteristics

The patients were also analyzed considering the previous use of BP: BP-naïve (*n* = 61) and BP-prior (*n* = 269); 95 patients were not included in this analysis because they had previously received teriparatide or strontium ranelate or due to insufficient data about previous treatments. The duration of previous BP treatment was 5.58 ± 0.28 years. There were no significant differences in BMI, years of menopause, serum calcium, urinary calcium, serum phosphate, 25(OH)D, and iPTH between BP-prior and BP-naïve (data not shown). Only age showed significant differences: BP-prior: 68.60 ± 0.58 years; BP-naïve: 66.62 ± 0.58 years (Mann-Whitney test, *p* = 0.0002).

#### 3.4.2. Bone Markers

As expected, basal BGP—but not tAP—was significantly lower in the BP-prior group due to previous antiresorptive treatment. In addition, s-CTX also was significantly lower in the BP-prior group versus BP-naïve group (Table 2).

After 1 year of treatment with Dmab tAP, BGP, and s-CTX were significantly decreased in both groups (Table 2).

#### 3.4.3. Bone Mineral Density

The increase in BMD after 1 year of treatment with Dmab in the whole group at the LS, FN, and TH was also found among both BP-naïve and BP-prior patients (Wilcoxon's signed rank test, *p* < 0.0001) (Table 3). However, the change in BMD was not different between BP-prior and BP-naïve (Mann-Whitney test, *p* > 0.05): LS: BP-prior: 5.46 ± 0.52%; BP-naïve: 5.31 ± 0.70%; FN: BP-prior: 4.22 ± 0.49%; BP-naïve: 3.97 ± 0.74%; TH: BP-prior: 2.85 ± 0.37%; BP-naïve: 3.07 ± 1.09% (Table 3).

### 3.5. Dmab Treated Patients Previously Treated with BP (BP-Prior) versus BP Treated Patients Not Switched to Dmab

There were no significant differences in main characteristics such as age, BMI, years of menopause, serum calcium, urinary calcium, serum phosphate, 25(OH)D, and iPTH between BP-prior group (*n* = 269) and control group (*n* = 148) (data not shown).

Also, both groups were similar in the duration of previous BP treatment (BP-prior: 5.58 ± 0.28 years; control group: 5.26 ± 0.26 years) and similar treatment regimens (BP-prior: 58.7% only one BP and 41.3% switched to another BP; BP-prior: 62.5% only one BP and 37.5% switched to another BP). In patients who received only one BP, the type of BP used was also similar (BP-prior: 42.2% ibandronate 150 mg/month, 18.8% alendronate 70 mg/week, 15.6% risedronate 150 mg/month, and 23.4% zoledronate 5 mg/year; control group: 42.7% ibandronate, 19.5% alendronate, 17.1% risedronate, and 20.7% zoledronate at the same doses) and in patients who were switched to other BP there was also similarity: while in the BP-prior group 40% of patients were switched to another oral BP and 60% to an intravenous BP, in the control group 42.2% were switched to another oral BP and 57.8% to an intravenous BP.

In contrast to the BP-prior group, the control group showed no significant differences in BGP and tAP in the last year of BP treatment. Although the control group showed a significant decrease in s-CTX, the percentage of change (↓25.98%) was lower than in the BP-prior group (↓67.86%) (Table 2).

Finally, when BMD was analyzed, a better response was observed in Dmab treated patients previously treated with BP compared with the control group, in whom significant increase was observed only in lumbar spine BMD, without significant differences in FN and TH BMD in the last year of BP treatment (Table 3).

## 4. Discussion

The present study evaluated the effect of Dmab on BMD and bone turnover markers after 1 year of treatment in clinical practice conditions. A good response in BMD was observed after 1 year of Dmab treatment at all studied regions: LS: +5.21%, FN: +3.50%, and TH: +2.54%. The reported effect of Dmab on BMD after 12 months is consistent with results from the FREEDOM trial, in which subjects were required to be off BP for 12 months prior to the study [2]. Also, these percentages of change were higher than those observed in a previous study where we evaluated the effect of strontium ranelate on BMD in a similar group of patients (LS: 3.73%, FN: 2.00%, and TH: 1.54%) [13]. Coincident with Dmab's mechanism of action a decrease of markers of bone formation (tAP and BGP) and bone resorption (s-CTX) was observed in the current study. The decrease of bone markers is coincident with data of dynamic histomorphometry in bone biopsies where the indices of bone turnover tended to be lower in the Dmab group than in placebo or alendronate groups [14]. In that study, median eroded surface was reduced by more than 80% and osteoclasts were absent from more than 50% of biopsies in the Dmab group. Double labeling in trabecular bone was observed in 94% of placebo bones and in 19% of those treated with Dmab. Meanwhile, double labeling in trabecular bone was present in 20% of the Dmab biopsies and in 90% of the alendronate samples. The significantly greater increases in BMD and reduction in s-CTX with Dmab compared to oral BP suggest that a more profound decrease of bone remodeling is achieved with Dmab than with oral BP.

The effect of Dmab on trabecular, subcortical, and cortical bone structure can explain the reduction in vertebral and nonvertebral fracture risk [15, 16]. Moreover, the decrease of cortical porosity induced by Dmab may contribute to increase of bone strength estimated by high-resolution peripheral quantitative computed tomography (HRpQCT) or by finite element analysis (FEA) of quantitative computed tomography (QCT) [17–19].

Poor adherence to antiosteoporotic medication has been associated with a significantly increased risk of fracture [20]. Improving medication adherence leads to a greater reduction in fracture rates after 2 years. It has been shown that adherence to Dmab was significantly greater (92.5%) than adherence to weekly alendronate (63.5%) [21].

In this study, when BMD (lumbar spine, femoral neck, and total hip) was analyzed considering the previous use of BP, a good response in both BP-naïve and BP-prior patients was observed. Previous studies have demonstrated that Dmab is effective in patients who have previously received BP [10–12]. Significantly greater BMD gains in patients transitioning to Dmab compared with subjects continuing on alendronate were achieved at 12 months at hip, lumbar spine, femoral neck, and 1/3 radius [11]. In our study, compliance with BP in the BP-prior group is suggested by significantly lower baseline BGP and s-CTX levels in the former. On the contrary, treatment with strontium ranelate in a similar population obtains better responses in BP-naïve groups [13, 22].

Although there are no studies in which the primary end point was to investigate the effects of prior-BP exposure on the treatment response to Dmab, comparing it to the response in BP-naïve patients, other studies have shown slightly larger BMD increments at the spine (2.5–3.0% in average) and hip (1% in average) among BP-naïve patients after Dmab treatment [2] than among patients switched to Dmab after BP treatment [11, 12]. Our findings showed no significant differences between BP-prior and BP-naïve patients in all studied regions. In addition, although this study is not prospective, a better densitometric response in Dmab treated patients previously treated with BP was observed compared with the control group, at all sites. According to this, in a recent publication in postmenopausal women with osteoporosis previously treated with oral BP, Dmab was associated with greater BMD increases and greater inhibition of bone remodeling compared with zoledronate [23].

It is important to note that the occurrence of adverse events and serious adverse events is similar after Dmab or monthly oral BP treatment [10]. In our study, no patient had to interrupt treatment with Dmab due to adverse effects.

Our study has limitations: it was not a prospective study, and the number of the BP-naïve women was less than that of BP-prior women; BMD and bone markers were recorded after 1 year of treatment without intermediate measurements, and measurements were not made in the same place and by the same person, although the same methods were used. Finally, it should be considered that most of the women in the BP-prior group were switched to Dmab because of poor clinical response to BP.

In conclusion, Dmab treatment increased BMD and decreased bone turnover markers in the whole group with similar response in BP-naïve and BP-prior patients. Also, a better BMD response in BP-prior patients versus BP treated patients not switched to Dmab was observed.

## Figures and Tables

**Table 1:** Baseline clinical characteristics of all patients (*n* = 425).

	Baseline
Age (years)	67.72 ± 0.48
Body mass index (kg/m^2^)	24.23 ± 0.25
Serum calcium (mg/dL)	9.46 ± 0.02
Urinary calcium (mg/24 h)	169.00 ± 6.48
Serum phosphate (mg/dL)	3.84 ± 0.03
25(OH)vitamin D (ng/mL)	33.98 ± 0.73
iPTH (pg/mL)	46.80 ± 1.01
tAP (IU/L)	149.40 ± 5.65
BGP (ng/mL)	19.33 ± 0.75
s-CTX (ng/L)	332.40 ± 14.39
Lumbar spine BMD (g/cm^2^; *T*-score)	0.864 ± 0.006; −2.70 ± 0.06
Femoral neck BMD (g/cm^2^; *T*-score)	0.742 ± 0.006; −2.38 ± 0.05
Total hip BMD (g/cm^2^; *T*-score)	0.747 ± 0.006; −2.17 ± 0.05

**Table 2:** Bone markers in patients treated with Dmab (BP-naïve and BP-prior) and BP treated patients not switched to Dmab (control group).

	Basal	12 m	Change (%)
*BP-naïve*			
tAP (IU/L)	160.60 ± 16.32	113.50 ± 11.28^#^	↓29.33
BGP (ng/mL)	26.78 ± 3.15^&^	20.22 ± 3.11^#^	↓24.50
s-CTX (ng/L)	509.80 ± 54.66^&^	101.40 ± 30.55^#^	↓80.11

*BP-prior*			
tAP (IU/L)	146.00 ± 010.21	117.90 ± 8.32^*∗*^	↓19.25
BGP (ng/mL)	19.58 ± 1.06	10.53 ± 0.65^*∗*^	↓46.22
s-CTX (ng/L)	314.90 ± 17.47	101.20 ± 9.00^*∗*^	↓67.86

*Control group*			
tAP (IU/L)	147.90 ± 7.01	141.30 ± 6.63	ns
BGP (ng/mL)	17.37 ± 2.00	16.08 ± 1.28	ns
s-CTX (ng/L)	275.60 ± 28.67	204.00 ± 17.05^*θ*^	↓25.98%

^#^Significant differences with BP-naïve basal; ^&^significant differences with BP-prior basal; ^*∗*^significant differences with BP-prior basal; ^*θ*^significant differences with control group basal.

**Table 3:** BMD in patients treated with Dmab (BP-naïve and BP-prior) and BP treated patients not switched to Dmab (control group).

	Basal	12 m	Change (%)
*BP-naïve*			
LS BMD (g/cm^2^)	0.866 ± 0.017	0.912 ± 0.018^#^	↑5.31
FN (g/cm^2^)	0.780 ± 0.017	0.811 ± 0.018^#^	↑3.97
TH (g/cm^2^)	0.749 ± 0.015	0.772 ± 0.014^#^	↑3.07

*BP-prior*			
LS BMD (g/cm^2^)	0.861 ± 0.008	0.908 ± 0.008^*∗*^	↑5.46
FN (g/cm^2^)	0.735 ± 0.008	0.766 ± 0.009^*∗*^	↑4.22
TH (g/cm^2^)	0.736 ± 0.006	0.757 ± 0.007^*∗*^	↑2.85

*Control group*			
LS BMD (g/cm^2^)	0.906 ± 0.009	0.923 ± 0.010^*θ*^	↑1.88%
FN (g/cm^2^)	0.745 ± 0.009	0.752 ± 0.008	ns
TH (g/cm^2^)	0.756 ± 0.012	0.758 ± 0.013	ns

^#^Significant differences with BP-naïve basal; ^*∗*^significant differences with BP-prior basal; ^*θ*^significant differences with control group basal.
